# Dispersal dynamics and introduction patterns of SARS-CoV-2 lineages in Iran

**DOI:** 10.1093/ve/veaf004

**Published:** 2025-01-27

**Authors:** Emanuele C Gustani-Buss, Mostafa Salehi-Vaziri, Philippe Lemey, Marijn Thijssen, Zahra Fereydouni, Zahra Ahmadi, Marc Van Ranst, Piet Maes, Mahmoud Reza Pourkarim, Ali Maleki

**Affiliations:** Laboratory of Clinical and Epidemiological Virology, Rega Institute, Department of Microbiology, Immunology and Transplantation, KU Leuven, Herestraat 49, Post Box 1040, Leuven BE-3000, Belgium; COVID-19 National Reference Laboratory (CNRL), Pasteur Institute of Iran, Pasteur Ave., No. 69, Tehran 1316943551, Iran; Department of Arboviruses and Viral Hemorrhagic Fevers (National Reference Laboratory), Pasteur Institute of Iran, Pasteur Ave., No. 69, Tehran 1316943551, Iran; Laboratory of Clinical and Epidemiological Virology, Rega Institute, Department of Microbiology, Immunology and Transplantation, KU Leuven, Herestraat 49, Post Box 1040, Leuven BE-3000, Belgium; Laboratory of Clinical and Epidemiological Virology, Rega Institute, Department of Microbiology, Immunology and Transplantation, KU Leuven, Herestraat 49, Post Box 1040, Leuven BE-3000, Belgium; COVID-19 National Reference Laboratory (CNRL), Pasteur Institute of Iran, Pasteur Ave., No. 69, Tehran 1316943551, Iran; COVID-19 National Reference Laboratory (CNRL), Pasteur Institute of Iran, Pasteur Ave., No. 69, Tehran 1316943551, Iran; Laboratory of Clinical and Epidemiological Virology, Rega Institute, Department of Microbiology, Immunology and Transplantation, KU Leuven, Herestraat 49, Post Box 1040, Leuven BE-3000, Belgium; Laboratory of Clinical and Epidemiological Virology, Rega Institute, Department of Microbiology, Immunology and Transplantation, KU Leuven, Herestraat 49, Post Box 1040, Leuven BE-3000, Belgium; Laboratory of Clinical and Epidemiological Virology, Rega Institute, Department of Microbiology, Immunology and Transplantation, KU Leuven, Herestraat 49, Post Box 1040, Leuven BE-3000, Belgium; Health Policy Research Centre, Institute of Health, Shiraz University of Medical Sciences, Shiraz 71348-14336, Iran; Blood Transfusion Research Centre, High Institute for Research and Education in Transfusion, Hemmat Exp.Way, Tehran 14665-1157, Iran; COVID-19 National Reference Laboratory (CNRL), Pasteur Institute of Iran, Pasteur Ave., No. 69, Tehran 1316943551, Iran; Department of Influenza and Respiratory Viruses, Pasteur Institute of Iran, Pasteur Ave., Tehran 1316943551, Iran

**Keywords:** SARS-CoV-2, COVID-19, genomic epidemiology, dispersal dynamic, phylogenetic, Iran, variants, epidemic, pandemic, travel, transmission, preparedness, vigilance, surveillance

## Abstract

Understanding the dispersal patterns of Severe Acute Respiratory Syndrome Coronavirus-2 (SARS-CoV-2) lineages is crucial to public health decision-making, especially in countries with limited access to viral genomic sequencing. This study provides a comprehensive epidemiological and phylodynamic perspective on SARS-CoV-2 lineage dispersal in Iran from February 2020 to July 2022. We explored the genomic epidemiology of SARS-CoV-2 combining 1281 genome sequences with spatial data in a phylogeographic framework. Our analyses shed light on multiple international imports seeding subsequent waves and on domestic dispersal dynamics. Lineage B.4 was identified to have been circulating in Iran, 29 days (95% highest probability density interval: 21–47) before non-pharmaceutical interventions were implemented. The importation dynamics throughout subsequent waves were primarily driven from the country or region where the variant was first reported and gradually shifted to other regions. At the national level, Tehran was the main source of dissemination across the country. Our study highlights the crucial role of continuous genomic surveillance and international collaboration for future pandemic preparedness and efforts to control viral transmission.

## Introduction

The Severe Acute Respiratory Syndrome Coronavirus-2 (SARS-CoV-2) was first identified in December 2019 in Wuhan, China, and rapidly spread globally, resulting in unprecedented mortality and significant health and economic impact (Li et al. 2020, Wu et al. 2020). SARS-CoV-2 has consistently evolved throughout the pandemic, resulting in variants that may exhibit different characteristics compared to the original strain. Throughout the COVID-19 pandemic, numerous variants of SARS-CoV-2 have been reported globally.

According to the WHO, there were two main categories of SARS-CoV-2 variants: variants of concern (VOCs) and variants of interest that exhibit specific characteristics, such as elevated transmissibility, increased severity, and the capability to evade immune responses induced by natural infection or vaccination (Salehi-Vaziri et al. 2022). So far, five VOCs have been documented: (i) the Alpha variant, known as the B.1.1.7 pango-lineage, first identified in the UK in September 2020 (Hill et al. 2022), (ii) the Beta variant, or B.1.351, initially detected in South Africa in October 2020 (Tegally et al. 2021), (iii) the Gamma variant, or P.1, first identified in Brazil in November 2020 (Faria et al. 2021), (iv) the Delta variant, or B.1.617.2, first identified in India in October 2020 (Mlcochova, 2021), and the (v) Omicron variant, designated as B.1.1.529, which emerged in Southern Africa in late November 2021 (Viana et al. 2022).

Iran was one of the first nations, apart from mainland China, to officially report outbreaks of SARS-CoV-2 in February 2020. As of 15 April 2023, the country has recorded 7 627 186 cases and 146 811 deaths (WHO 2024). Understanding the dispersal patterns of SARS-CoV-2 variants during epidemic waves is crucial for future public health interventions, such as establishing a robust surveillance system and an effective preparedness program. However, a comprehensive understanding of the genomic epidemiology of SARS-CoV-2 in Iran throughout the various epidemic waves is still lacking, in particular how variants were introduced and replaced by new variants in subsequent waves. Furthermore, it is crucial to deepen our knowledge about the regions that have contributed to the viral transmission to Iran, likely facilitated by international air mobility.

In this study, we investigated the genomic epidemiology of SARS-CoV-2 in Iran from 20 February 2020 to 16 July 2022. Using a discrete phylogeographic approach, we aimed to identify the sources of transmission and trace the viral dynamics at both global and local levels for the B.4 lineage, and the Alpha, Delta, and Omicron VOCs. Our findings contribute to a more comprehensive understanding of the imports, exports, and the dynamic dispersal processes of these variants from the initial days of the pandemic up to two years of virus dissemination.

## Methods

### Sample collection

In accordance with the protocol of our ethically approved project by the Pasteur Institute of Iran (approval code: IR.PII.REC.1399.073), oro/naso-pharyngeal swab samples were collected from individuals diagnosed with SARS-CoV-2 across various provinces of Iran spanning the period from 20 February 2020 to 16 July 2022. These collected samples were preserved in viral transport medium and were subsequently shipped to the COVID-19 National Reference Laboratory (CNRL) at the Pasteur Institute of Iran for continuous genomic surveillance of SARS-CoV-2 with a particular focus on understanding the origin of surges in cases. Genomic sequencing of SARS-CoV-2 encompassed 1281 samples, constituting a fraction of specimens that met the required qualifications. The inclusion criteria of the present study were as follows: (i) Iranian nationality, (ii) suitable viral load for sequencing [cycle threshold (CT) values <25], and (iii) complete necessary metadata including date of sample collection, date of symptom onset, location, age, and sex.

### Viral genomic RNA isolation

In a biological safety level 2 plus condition, viral nucleic acid was extracted using the Nucleic Acid Extraction Kit (Zybio, China) and employing the automated nucleic acid isolation system EXM3000 (Zybio, China) as previously described (Ahmadi et al. 2023). Briefly, 200 µl of sample and 15 µl of proteinase K were added to the 96-well plate and nucleic acid was allowed to bind to magnetic beads. After washing away any unbound samples, the RNA was eluted and stored at −80°C until further analyses including reverse transcription–polymerase chain reaction (RT-PCR) to confirm the presence of SARS-CoV-2.

### SARS-CoV-2 real-time PCR

The extracted RNA was assessed for SARS-CoV-2 using the 2019-nCoV nucleic acid diagnostic kit (Sansure Biotech, China) as previously described (Salehi-Vaziri et al. 2021). The samples underwent reverse transcription at 50°C for 30 min, followed by primary denaturation at 95°C for 1 min. The subsequent stage consisted of 45 cycles of secondary denaturation for 15 s at 95°C and annealing/extension for 30 s at 60°C, facilitated by the Qiagen Rotor-Gene Q (Qiagen, Germany).

### Next-generation sequencing by Oxford Nanopore Technologies

To determine the appropriateness of the samples for next-generation sequencing (NGS), filtering was conducted using a reverse transcription–quantitative polymerase chain reaction assay, with a specific focus on CT values (not exceeding 25 for the N and ORF1ab genes). Subsequently, library preparation for NGS libraries were prepared using Midnight RT-PCR Expansion (EXP-MRT001) and the rapid barcoding SQK-RBK110.96 kit from Oxford Nanopore Technologies (ONT, UK) as previously described (Ahmadi et al. 2023).

The NGS library preparation process commenced with complementary DNA synthesis using LunaScript RT SuperMix (NEB, USA). Subsequently, the whole genome was amplified using Q5 HS Master Mix (NEB, USA), together with two sets of primers known as Midnight Primer Pool A and Midnight Primer Pool B. Following the amplification process, the individual PCR products were combined, and their concentration was determined using the Qubit dsDNA HS Assay kit and Qubit 4 Fluorometer (ThermoFisher Scientific, USA). The highest quality PCR products were then carefully selected and assigned a barcode with the Rapid Barcode Plate RB96 (ONT, UK), to ensure accurate identification throughout the remaining steps of the NGS library preparation. After successful barcoding, the final pool of the NGS library underwent a thorough cleanup procedure using specialized solid-phase reversible immobilization beads (ONT, UK). The cleaned-up library was then ligated with Rapid Adapter F, marking the conclusion of the NGS library preparation process.

Finally, the prepared library was loaded onto the Flow Cell (R9.4.1, ONT, UK) and analyzed using the ONT GridION machine (ONT, UK). Base-calling, quality control of FASTQ data, variant calling, and FASTA generation were performed using the MinKNOW software (ONT, UK).

### Phylogenetic inference

The SARS-CoV-2 genomic data collected in Iran were assigned to lineages and clades using the Phylogenetic Assignment of Named Global Outbreak Lineages (pangolin) tool v4.2 (O’Toole, 2021) and NextClade (v3.2.0) (Aksamentov et al. 2021), respectively.

To investigate the dynamics of the SARS-CoV-2 lineages in a global context, we downloaded high-quality complete genome sequences from the Global Initiative on Sharing All Influenza Data (GISAID) database (https://gisaid.org/) to construct specific background databases for the analysis of each lineage. An Augur subsampling strategy (Huddleston et al. 2021) considering region, year, and month of sampling was applied to different collections of VOCs: Alpha (*n* = 16 646, before subsampling), Delta (*n* = 185 615), and Omicron (*n* = 1 048 576) (Huddleston et al. 2021). For the B.4 lineage, all sequences available on GISAID were retrieved resulting in 555 genomes collected between 18 January 2020 and 16 July 2020 from Africa (*n* = 3), Asia (*n* = 131), Europe (*n* = 70), Iran (*n* = 287; this study: *n* = 253; GISAID: *n* = 34), North America (*n* = 35), and Oceania (*n* = 29). The Alpha variant dataset was composed of 1227 sequences from Africa (*n* = 164), Asia (*n* = 145), Europe (*n* = 280), Iran (*n* = 179; this study: *n* = 86; GISAID: *n* = 93), North America (*n* = 171), Oceania (*n* = 129), and South America (*n* = 159), encompassing samples between 9 September 2020 and 30 October 2021. The Delta variant dataset consisted of 1099 sequences from Africa (*n* = 143), Asia (*n* = 186), Europe (*n* = 169), Iran (*n*= 203; this study: *n* = 195; GISAID: *n* = 8), North America (*n* = 176), Oceania (*n* = 116), and South America (*n* = 106). For the Omicron variant, filtering using the Augur approach (Huddleston et al. 2021) resulted in 653 genomes representing the global context, amounting to 1228 sequences collected from 2 November 2021, until 16 July 2022, from Africa (*n* = 56), Asia (*n* = 131), Europe (*n* = 320), Iran (this study: *n* = 575), North America (*n* = 70), Oceania (*n* = 20), and South America (*n* = 56) (see Supplementary File S2).

We used Nextalign CLI/Nextrain (v2.0.0) (Hadfield et al. 2018) for multiple sequence alignment and Aliview (Larsson, 2014) for trimming and visual inspection. We used IQTREE2 (v.2) (Minh et al. 2020) to reconstruct a maximum likelihood phylogenetic tree, using a general time-reversible model with empirical base frequencies and gamma-distributed rate heterogeneity across sites (with four categories), which was selected as the best-fitting substitution model by the model finder option implemented in this software. The branch support was estimated based on an Shimodaira–Hasegawa-like approximate likelihood ratio test with 1000 replicates. We used a root-to-tip regression approach in TempEst version 1.5.3 (Rambaut et al. 2016) to evaluate the temporal or molecular clock signal in the phylogeny. The assessment was conducted by regressing root-to-tip divergence as a function of sampling dates, with outliers removed using the interquartile range method.

### Demographic and phylogeographic reconstruction

To reconstruct the spread of each SARS-CoV-2 lineage in a global context, genomes were assigned to the global regions (Africa, Asia, Europe, North America, South America, and Oceania). We used a strict clock model, with a fixed rate (8 × 10^−4^ substitutions per site per year), and a Bayesian Skygrid (Gill et al. 2013) coalescent tree prior with weekly time intervals, implemented in Beast 1.10.4 (Suchard et al. 2018) and using the BEAGLE library (Ayres et al. 2019) for parallelization of the likelihood calculations. The skygrid models with weekly intervals included 27, 48, 55, and 31 grids for the B.4, Alpha, Delta, and Omicron datasets, respectively. Subsequently, for a set of 1000 posterior trees, we performed a discrete trait ancestral reconstruction using an asymmetric model with a prior on the total number of included rates recommended by Gao et al. (2023). In addition, we used a Bayesian stochastic search variable selection method to quantify the support of migration routes using Bayes factors. We implemented Markov jumps (MJs) counts to infer the number of transitions between each region and Iran (Lemey et al. 2014). Additionally, we used MJs to summarize the timing of the earliest transitions between location states based on 1000 posterior trees to characterize introduction patterns. To infer statistical support for lineage transition events, we computed the Bayes factors for B.4 and for the VOCs (Alpha, Delta, and Omicron). We report MJs associated with transition rates that yield a BF support ≥ 10. Two independent Markov Chain Monte Carlo simulations were run for 100 million iterations sampling every 50 000 steps for all lineages evaluated here. We used Tracer 1.7.2 (Rambaut et al. 2018) to evaluate the convergence of posterior estimates, considering effective sample sizes > 200, and to generate a Bayesian Skygrid reconstruction plot. In addition, we used TreeAnnotator (Suchard et al. 2018) discarding 10% as burnin-in to summarize Maximum Clade Credibility (MCC) trees. To inspect the root-state posterior probability, we used FigTree v.1.4.4 (Rambaut et al. 2018) to visualize the tree with annotations and the ggtree R package (Yu 2020). The demographic trajectory for Iranian clades was inferred using a skygrid multilocus approach for those clades containing at least 20 sequences.

Viral migration estimates using MJs and their timing were collected from the posterior Tree distribution using the TreeMarkovJumpHistoryAnalyzer tool (Lemey, 2021) and plotted using the Circlize R package (Gu et al. 2014). To characterize within-country transmission, we considered 5 administrative regions by the Ministry of Interior grouping the 31 provinces (De Rosi, 2020). The division consists of Region 1 (capital city, Tehran: *n* = 525), Region 2 (capital city, Isfahan: *n* = 140), Region 3 (capital city, Tabriz: *n* = 108), Region 4 (capital city, Kermanshah: *n* = 143), and Region 5 (capital city, Mashhad: *n* = 317). To reconstruct viral dispersal between these regions, we performed a Bayesian discrete diffusion analysis following all the steps described above.

## Results

### The dynamics of SARS-CoV-2 waves in Iran

**Figure 1.**
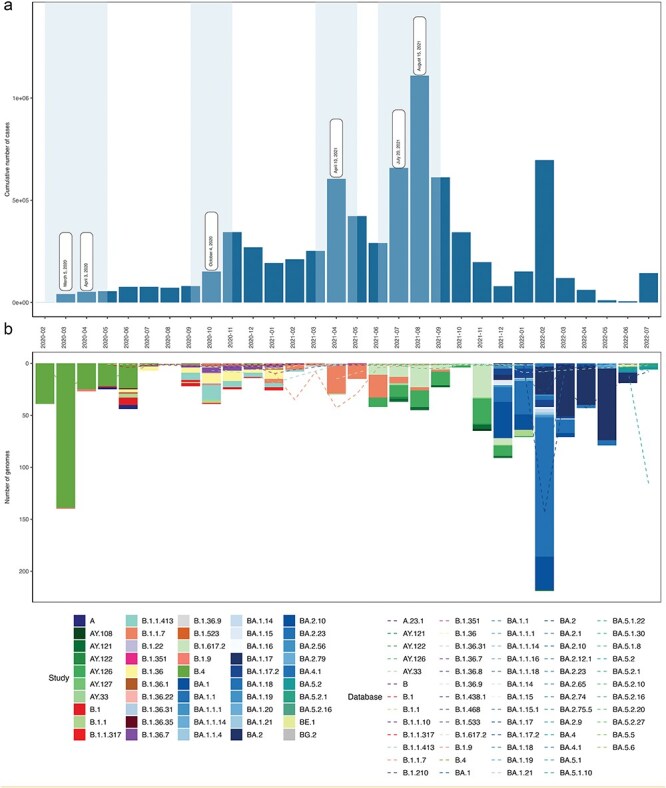
Epidemiological landscape of SARS-CoV-2 genomes collected in Iran between 2 February 2020 and 7 July 2022.(a) Histogram of cumulative number of cases accounted per month and white boxes and light blue represent the timeline of lockdown events during the waves in Iran (Our World in Data, Mathieu et al. 2020); (b) Histogram of number of genomes sampled and characterized during waves sampled. The dashed lines correspond to sequences available on GISAID.

Based on the sequences sampled (*n* = 1281) across six waves of COVID-19 in Iran (Fig. 1a, confirmed cases based on Our World in Data: (Mathieu et al. 2020), the epidemiological landscape underwent dramatic changes between 20 February 2020 and 16 July 2022. The first six waves follow the description by Amin et al. (2022). The first case report on 19 February marked the start of the initial wave that exhibited a substantial increase in case numbers from 388 cases to a peak of 41 000 new cases on 31 March 2020 (Fig. 1a) (Mathieu et al. 2020). This surge was predominantly fueled by the B.4 lineage, marking a period of substantial community transmission, and prompting the implementation of lockdowns as a measure to reduce cases and deaths (Fig. 1a and b). The second wave of the pandemic in Iran was driven by the surge composed of lineages such as B.4, B.1.1, B.1.9, and B.1.1.413, and B.1.36, resulting in 80 067 new cases by July 2020 (Fig. 1 and Table 1), while the third wave was marked by a peak in November 2020, a total of 151 315 cases, and the circulation of lineages B.1.1.413, B.1.36, and B.1.36.7.

**Table 1.** Summary of lineage diversity and clades collected for Iran study.

Lineages	Nextclade	%
A	19B	0.005 (6)
B.1	20B	0.012 (15)
B.1.1	20B	0.013 (16)
B.1.1.317	20B	0.005 (6)
B.1.1.413	20B	0.030 (38)
B.1.1.7	20I (Alpha)	0.067 (86)
B.1.22	20A	0.001 (1)
B.1.351	20H (Beta)	0.001 (1)
B.1.36	20A	0.034 (43)
B.1.36.1	20A	0.001 (1)
B.1.36.22	20A	0.002 (2)
B.1.36.31	20A	0.001 (1)
B.1.36.35	20A	0.001 (1)
B.1.36.7	20A	0.016 (21)
B.1.36.9	20A	0.001 (1)
B.1.523	20A	0.001 (1)
B.1.9	20A	0.008 (10)
B.4	19A	0.198 (253)
B.1.617.2	21A (Delta)	0.005^5^ (7)
B.1.617.2	21J (Delta)	0.067^5^(87)
AY.108	21J (Delta)	0.002 (2)
AY.121	21J (Delta)	0.012 (15)
AY.122	21J (Delta)	0.002 (3)
AY.126	21J (Delta)	0.067 (86)
AY.127	21J (Delta)	0.001 (1)
AY.33	21J (Delta)	0.002 (2)
BA.1	21K (Omicron)	0.067 (86)
BA.1.1	21K (Omicron)	0.157 (201)
BA.1.1.1	21K (Omicron)	0.003 (4)
BA.1.1.14	21K (Omicron)	0.002 (3)
BA.1.1.4	21K (Omicron)	0.002 (3)
BA.1.14	21K (Omicron)	0.003 (4)
BA.1.15	21K (Omicron)	0.005 (7)
BA.1.16	21K (Omicron)	0.001 (1)
BA.1.17	21K (Omicron)	0.005 (7)
BA.1.17.2	21K (Omicron)	0.017 (22)
BA.1.18	21K (Omicron)	0.005 (7)
BA.1.19	21K (Omicron)	0.003 (4)
BA.1.20	21K (Omicron)	0.001 (1)
BA.1.21	21K (Omicron)	0.001 (1)
BA.2	21L (Omicron)	0.153 (196)
BA.2.10	21L (Omicron)	0.002 (2)
BA.2.23	21L (Omicron)	0.003 (4)
BA.2.56	21L (Omicron)	0.002 (2)
BA.2.79	21L (Omicron)	0.002 (3)
BA.4.1	22A (Omicron)	0.001 (1)
BA.5.2	22B (Omicron)	0.005 (7)
BA.5.2.1	22B (Omicron)	0.002 (3)
BA.5.2.16	22B (Omicron)	0.001 (1)
BE.1	22B (Omicron)	0.002 (2)
BG.2	22C (Omicron)	0.001 (1)
XE	Recombinant	0.001 (1)

The introduction of the first VOC, Alpha (B.1.1.7), in Iran led to a rapid expansion of cases during the fourth wave, reaching 604 571 reported cases and peaking in April 2021. The fifth wave, which started in July and peaked in August 2021, was mainly driven by the Delta variant, and accounted for 1.1 million reported cases. This was followed by the sixth wave that was primarily caused by Omicron. The high number of Omicron cases (696 288 reported, Fig. 1a and b) coincided with the relaxation of social restrictions, including the lifting of social distancing and flight bans, despite ongoing vaccination efforts (Fig. 1a and b).

In this study, we identified a total of 52 distinct lineages throughout the various waves of the pandemic. Most isolates belonged to B.4 (19A, *n* = 253, 19.8%) from the first wave. Subsequently, B.1.1.7 (20I Alpha, *n* = 86, 6.7%), B.617.2 (21 J Delta, *n* = 87, 6.75%), and AY.126 (21 J Delta, *n* = 86, 6.7%) were also among the frequently observed lineages during the fourth and fifth wave. BA.1.1 (21K Omicron, *n* = 201, 15.7%) and BA.2 (21L Omicron, *n* = 196, 15.3%) accounted for the most commonly observed lineages in the subsequent waves, in particular in the sixth wave (Fig. 1b and Table 1). All sequences generated throughout this study have been deposited into the GISAID (see Supplementary File S1).

### Bayesian reconstruction of discrete spatial diffusion

**Figure 2.**
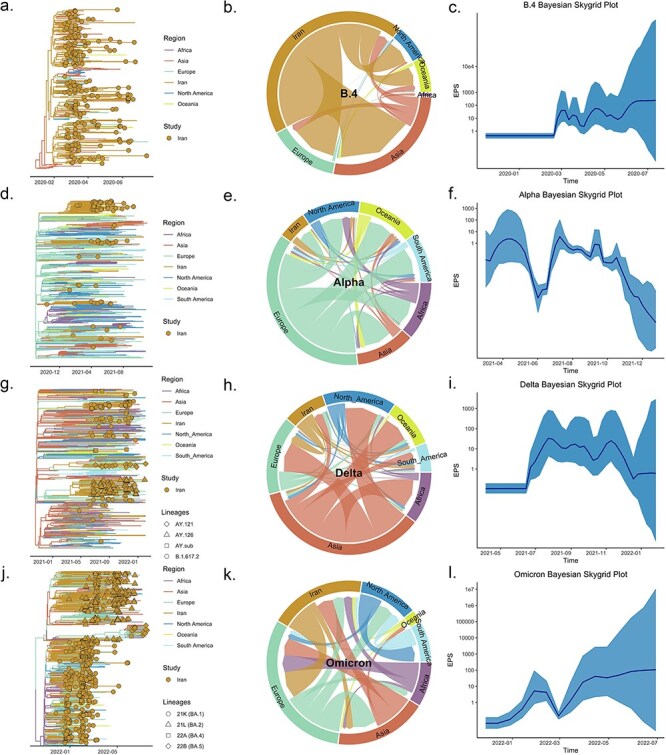
Phylogeographic reconstruction of SARS-CoV-2 lineages responsible for major epidemic waves in Iran. The MCC trees (left panel) are color-coded based on the estimated or sampled region while shapes represent sublineages for Delta and Omicron. The flows of migration among all regions during four waves in Iran are summarized by circular plots, with arrowheads indicating the start and end points (central panel). Bayesian Skygrid plots reconstructed for the Iranian transmission clades (right panel). (a–c): b.4, (d–f): Alpha, and (g–i): Delta.

**Figure 3.**
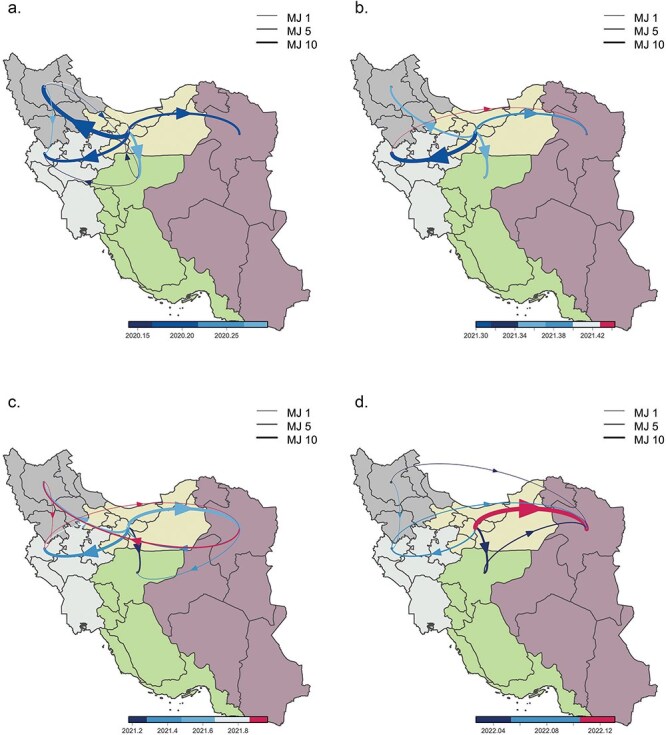
Dispersal dynamics among sampled SARS-CoV-2 lineages across five regions in Iran. The discrete phylogeographic reconstruction traces the dispersal history of viral lineages, encompassing the B.4 (a) lineage and the Alpha (b), Delta (c), and Omicron (d) variants. In the discrete reconstructions, we present the number of lineage dispersal movements estimated by MJs between regions (depicted by arrows), extracted from trees sampled in each posterior distribution and color-coded by time interval. The arrow thickness is proportional to the number of MJ events. Only MJ events with an adjusted Bayes factor support higher than 10 are included.

We used a Bayesian discrete phylogeographic approach to reconstruct the migration dynamics of B.4, which circulated extensively in early 2020 and led to a peak in cases in Iran. Our reconstructions indicate introductions from Asia (Fig. 2a, left panel) preceding B.4 diversification, with the first introduction estimated to have occurred around 19 January 2020 [95% highest probability density (HPD): 4 January 2020–30 January 2020]. This indicates a potential lag of 29 days (95% HPD interval: 21–47) between detection and viral introduction, which suggests that the introduction from Asia to Iran took place preceding the implementation of international flight restrictions in late January 2020 (Fig. 3). The phylogeographic reconstructions revealed a total of eight introductions (95% HPD interval: 1–16), mainly from Asia. In addition, we identified exports from Iran to Asia (MJ = 59, 95% HPD interval: 50–69), to Europe (MJ = 34, 95% HPD interval: 29–38), North America (MJ = 18, 95% HPD interval: 0–40), and to Oceania (MJ = 13, 95% HPD interval: 10–17) (Fig. 2b). Those events suggest that Iran could have served as one of the potential sources of spread to other geographical regions such as Europe, North America, and Oceania. Estimates of the B.4 effective population size through time in Iran indicate epidemic growth beginning in March 2020, and irregular dynamics with no clear decline by July 2020, in line with the co-circulation of this lineage in the second wave together with lineages B.1.1, B.1.9, and B.1.1.413 (Fig. 2c).

While the Alpha variant (B.1.1.7) was already circulating together with lineages from the third wave (Fig. 1b), it was almost solely responsible for the fourth epidemic wave in Iran. Our results point at multiple independent introductions of this variant, resulting in two large clades with predominantly samples from Iran, reflecting intense circulation within the country (Fig. 2d). We estimate the earliest introduction to have occurred from Europe on 24 September 2020 (95% HPD: 9 September–8 October 2020). This implies a reporting lag of 20 days (95% HPD: 6–35) before the first sampled Alpha sequence in Iran on 14 October 2020, originating from a patient with international travel history through countries in Europe and Asia. The phylogeographic reconstruction of our sample revealed a total of 18 introductions (95% HPD interval: 15–22), from Europe. Furthermore, four exports (95% HPD interval: 2–5) were identified to North America as well as four exports (95% HPD interval: 3–6) to Oceania (Fig. 2e). The demographic reconstruction for the major Iranian clades suggests an expansion starting in early 2021, reaching a peak in April 2021 (Fig. 2b), and after a decline in June 2021, relatively diverse Alpha lineages remained co-circulating with the Delta variant during the fifth wave.

The lineages classified within the Delta variant were observed to circulate extensively between June and December 2021, contributing to a notable surge in cases at the onset of the fifth wave in August 2021, despite the implementation of social distance measures (Fig. 1a and b). Phylogeographic analyses suggested that three main lineages were responsible for community transmission (AY.121, AY.126, and B.1.617.2, respectively) (Fig. 2g), primarily linked to viral introductions from Europe and Asia. The importation scenario corresponded to main sources from Europe starting on 13 February 2021 (95% HPD: 21 December 2020–14 April 2021) and followed by an import from Asia starting on 28 February 2021 (95% HPD: 28 December 2020–16 April 2021). The phylogeographic inference for our sample estimated a total of 16 introductions (95% HPD interval: 12–20) from Asia to Iran, 7 events from Europe (95% HPD interval: 4–10), and 33 exports (18, 95% HPD interval: 15–21) to Europe (4, 95% HPD interval: 2–6), to Asia (5, 95% HPD interval: 5–7), to South America (6, 95% HPD interval: 2–7), and to Oceania (5, 95% HPD interval: 2–7) (Fig. 2h). The Bayesian Skygrid reconstruction indicated an expansion in effective viral population size beginning in mid-2021 to a level that was roughly maintained until the end of 2021 (Fig. 2i).

Omicron variants seeded the sixth wave and caused a rise in cases in early 2022 (Table 1 and Fig. 1a and b). Our phylodynamic reconstruction unveiled multiple introductions from African and European countries and a phylogenetic division in two Iranian clades. The largest clade contains mostly sequences classified as BA.1, while the second largest clade is predominantly composed of BA.2 sequences (15.3%). The earliest introduction from Africa was estimated around 7 November 2021 (95% HPD: 12 October 2021–25 November 2021) (Fig. 2k) and from Europe on 18 November 2021 (95% HPD: 27 October 2021–10 December 2021), which coincide with the relaxation of intervention measures. This translates to an average detection lag of 19 days for Omicron (95% HPD: 1–44 days).

The phylogeographic analysis uncovered multiple introductions from Africa (43, 95% HPD interval: 12–76), Asia (81, 95% HPD interval: 27–150), and Europe (60, 95% HPD interval: 5–120), as well as multiple exports to Europe (132, 95% HPD interval: 90–177). The observation of a heterogeneous source is consistent with global dissemination fueling Omicron transmission when flight restrictions were globally relaxed (Chinazzi et al. 2020; Tegally et al. 2023). The effective population size estimates indicate an initial increase in early 2022, followed by a decline and subsequent rise in cases by April 2022 (Fig. 2l). This trend persisted and intensified in the subsequent months, as evidenced by the increasing number of cases (Fig. 1a).

### Phylogeographic spread across Iranian regions

To gain insights into the patterns of SARS-CoV-2 spread in Iran, we also conducted a Bayesian phylogeographic reconstruction at the within-country level between five major administrative regions. The majority of transitions were inferred from Region 1, which includes Tehran, to all other regions (Fig. 3). This observation underscores Tehran’s crucial role as a primary source for the dissemination of both VOCs and other lineages, displaying diverse transition patterns throughout the waves of the pandemic. The phylogeographic analysis for lineage B.4 revealed an initial transition from Tehran to Region 2 (Isfahan) on 29 January 2020 (95% HPD: 21 January 2021–6 February 2020) (Fig. 3a). In the case of the Alpha variant, the first transition from Tehran to Region 4 (Kermanshah) occurred on 26 November 2020 (95% HPD: 3 November 2021–1 December 2021) (Fig. 3b). For the Delta variant, the first transition took place on 16 April 2021 (95% HPD: 25 March 2021—8 May 2021) to Region 5 (Mashhad) (Fig. 3c). Also, for the Omicron variant, the initial export from Tehran was to Region 5 (Mashhad) on 27 November 2021 (95% HPD: 13 November 2021–7 December 2021) (Fig. 3d). Further examining the B.4 dynamics identified secondary fluxes from Region 2 (Isfahan) and Region 3 (Tabriz) to Region 4 (Kermanshah). In contrast, the pattern for Alpha sequences displayed different secondary flux patterns, with movement from Region 4 (Kermanshah) to Region 5 (Mashhad). For Delta and Omicron, secondary fluxes were inferred from Region 3 (Tabriz) and Region 4 (Kermanshah) to Region 5 (Mashhad).

## Discussion

Our study elucidated the diversity of SARS-CoV-2 lineages, B.4, Alpha, Delta, and Omicron, and their migration routes into Iran between 2020 and 2022. The dispersal pathways underscore multiple sources of four SARS-CoV-2 lineages, highlighting the interplay between global and local factors in driving the transmission dynamics of SARS-CoV-2 waves across the country. The findings indicated that new lineages were primarily introduced from the region where the variant was first reported, and gradually shifted to other regions as was particularly the case for the Omicron wave (Eden et al. 2020). These variants became widespread due to the progressive lifting of mobility restrictions as also indicated by previous work (McCrone et al. 2022).

The global introductions suggested that international air travel has likely played a crucial role in SARS-CoV-2 dissemination, in line with what has been reported previously as a key driver of viral propagation in several other studies (Lemey et al. 2020, Hodcroft et al. 2021, Viana et al. 2022; Tegally et al. 2023). Air travel includes tourism but also the repatriation of citizens and people seeking medical treatment, all of which can significantly influence the transmission dynamics and geographical distribution of the virus (Hale et al. 2021). Unfortunately, we did not have access to passenger numbers to formally assess its role as covariate in the dissemination dynamics.

For B.4, our findings support that after introduction from Asia, this variant spread extensively in Iran and subsequently to other regions, such as Europe and Oceania. The earliest report of lineage B.4 dates back to 18 January 2020, with initial cases identified in Wuhan and Shandong provinces, China. This suggests that the lineage emerged early and diversified during initial outbreaks, common to the B lineages that subsequently dominated human populations globally (Lv et al. 2024). The circulation of B.4 in Iran and exports to Oceania corroborates previous work that implicated B.4 transmission from Iran through travelers in late February 2020 (Lemey et al. 2020).

The fourth wave in Iran was attributed to the Alpha variant. We isolated an Alpha variant in October 2020 from a traveler returning home after a work trip to Europe and our inference suggested that the Alpha variant was circulating in Iran as early as mid-September 2020. This may represent somewhat of an overestimate given that the TMRCA of the Alpha clade in the UK was estimated at 28 August 2020 (95% HPD: 15 August 2020–9 September 2020) (Hill et al. 2022). It is, however, in broad agreement with evidence of silent spread of this lineage in Iran (Eslami et al. 2022, Rezaei et al. 2023). The international Alpha alert on 18 December 2020 prompted genomic surveillance globally as well as travel bans (Kraemer et al. 2021, O’Toole et al. 2021). Despite these measures, Alpha spread intensively in more than 60 countries, in many cases tracing back to an importation from the UK, sometimes occurring days or even months before official notification (Faucher et al. 2024). Our estimates for the introduction of Delta in Iran in February 2021 from Asia are considerably earlier than the first sequenced genome in late April of that year. The demographic estimates indicate that the variant only started to significantly grow in population size in June–July. This resulted in a significant surge in cases in Iran, peaking at over 1.1 million cases in August 2021. As for Delta, Omicron also caused a significant rise in case (Arantes et al. 2023). Our study identified BA.1 and BA.2 as the main Omicron lineages responsible for the sixth wave of the COVID-19 pandemic in Iran. Recent studies have identified several Omicron lineages, including BA.1, BA.1.1, BA.2, BA.4, and BA.5, as contributors to subsequent waves of COVID-19 infections around the world (Tegally et al. 2022, Chatterjee et al. 2023). These lineages were initially reported from Botswana and South Africa and quickly became the most frequently observed variants in various regions worldwide (Viana et al. 2022). While BA.1 dominated briefly, BA.2 rapidly emerged as the predominant variant in many countries (Hirotsu, 2022), owing to its higher transmissibility. Our reconstructions indicated Africa as the primary source for BA.1, followed by European countries as a source for BA.2 in November 2021. Our findings are therefore consistent with earlier studies that reported initial imports of the Omicron variant from Africa, which quickly shifted to European countries as a source during November and December 2021 (Torjesen, 2021, Tian et al. 2022). Those introductions resulted in increased transmission rates, particularly in late December, raising concerns about the potential for immune escape (Chen et al. 2023, Tegally et al. 2023). Low vaccination coverage in 2021 and limited reductions in mobility may also have contributed to extensive virus circulation of the Delta and Omicron variants. The vaccination campaign intensified on 20 August 2021, with 21 805 879 doses of various vaccines administered through consortium agreements. In September, 6% of the population was fully vaccinated, and by April 2022, it had increased to ∼65%–75% (fully vaccinated) (Mathieu et al. 2020, WHO, 2021). While vaccination was critical in preventing disease burden, non-pharmaceutical interventions also remained crucial in controlling spread (WHO 2021). However, the last official lockdowns were implemented in late August 2021. Subsequently, there was a progressive relaxation of measures such as social distancing, travel bans, and, finally, mask-wearing (Amin et al. 2022).

The country-level migration reconstructions pointed at the Tehran region (Region 1) as the primary source of spread for all lineages across different waves, accompanied by various secondary routes. This finding can be aligned with the fact that the capital city of Iran is densely populated, associated with the main international airport, has important health care infrastructure, and is a city to which millions of people from other cities commute on a daily basis. Therefore, Tehran played an important role in spreading SARS-CoV-2 in the country, a pattern that would likely be repeated in the next epidemic/pandemic (Tondelli et al. 2022, Zhang et al. 2022). Other regions that were identified as the primary and secondary sources play important roles as tourist destinations, serving as the key destinations for national and international flight passengers, healthcare services, and pilgrimage journeys during holidays. For example, in the early stages of the epidemic, significant transmission was attributed to New Year festivities (mid-March to half mid-April) and to the summer holiday period (June–September) in Gilan, Qom, and Golestan (Poustchi, 2021; Zhang et al. 2022, Khazaee-Pool et al. 2024).

While we capitalize on genomic data covering two years of surveillance efforts in Iran, an important limitation of our study is that the background data used to address the circulation dynamics were derived from publicly available genomic surveillance data and were subjected to a subsampling strategy to alleviate the high computational burden. Additionally, we have no good insights to what extent differences in sampling across regions in Iran reflect differences in case numbers.

## Conclusion

In conclusion, our study represents an effort to broaden our understanding of the main sources and dissemination dynamics of SARS-CoV-2 lineages in Iran between 20 February 2020 and 16 July 2022. The study sheds light on their imports, exports, the chronology of these events, and transmission patterns within Iran, as well as potential ramifications for public health strategies. The Bayesian reconstruction of discrete spatial diffusion provided insights into the geographical sources of B.4, Alpha, Delta, and Omicron lineages, revealing the contribution of Asia, North America, and European regions to the dissemination in Iran. Our results emphasize the importance of early warning systems as a crucial preparedness measure, enabling an effective response to outbreaks, which is needed to face potential health crises in the future.

## Supplementary Material

veaf004_Supp

## Data Availability

All metadata and genomes employed in this study are available on GISAID’s EPICoV database. The information for all sequences is enclosed as Supplementary Table 3. Beast XML files used in this work are publicly available on GitHub (https://github.com/emanuelegustani/dispersal_dynamic.git).
