# Supplementary material for: Dispersal dynamics and introduction patterns of SARS-CoV-2 lineages in Iran
Source: Virus Evol. 2025 Jan 27;11(1):veaf004. doi: 10.1093/ve/veaf004 (PMC11803630; doi:10.1093/ve/veaf004)
Supplement: veaf004_Supp [file veaf004_supp.zip › suppl_data/Supplementary material.docx]

**Supplementary material**

**Dispersal dynamics and introduction patterns of SARS-CoV-2 lineages in Iran**

Emanuele C. Gustani-Buss^1*^, Mostafa Salehi-Vaziri^2,3*^, Philippe Lemey^1*^, Marijn Thijssen^1^, Zahra Fereydouni^2^, Zahra Ahmadi^2^, Marc Van Ranst^1^, Piet Maes^1^, Mahmoud Reza Pourkarim^1,4,5*†^ and Ali Maleki^2,6*†^

^1^Department of Microbiology, Immunology and Transplantation, KU Leuven, Rega Institute, Laboratory of Clinical and Epidemiological Virology, Herestraat 49, 3000 Leuven, Belgium.

^2^COVID-19 National Reference Laboratory (CNRL), Pasteur Institute of Iran, Pasteur Ave., Tehran, Iran.

^3^Department of Arboviruses and Viral Hemorrhagic Fevers (National Reference Laboratory), Pasteur Institute of Iran, Pasteur Ave., Tehran, Iran.

^4^Health Policy Research Centre, Institute of Health, Shiraz University of Medical Sciences, Shiraz 71348-14336, Iran.

^5^Blood Transfusion Research Centre, High Institute for Research and Education in Transfusion, Hemmat Exp.Way, Tehran 14665-1157, Iran.

^6^Department of Influenza and Respiratory Viruses, Pasteur Institute of Iran, Pasteur Ave., Tehran, Iran.

**Contributed equally*

^†^*Correspondence to: Dr. Mahmoud Reza Pourkarim and Dr. Ali Maleki*

-Laboratory for Clinical and Epidemiological Virology

Department of Microbiology, Immunology and Transplantation, Herestraat 49, Post box 1040, BE-3000 Leuven, Belgium, KU Leuven, BE-3000 Leuven, Belgium

*Email:* [*Mahmoudreza.pourkarim@kuleuven.be*](mailto:Mahmoudreza.pourkarim@kuleuven.be)

*-COVID-19 National Reference Laboratory (CNRL), Pasteur Institute of Iran, Pasteur Ave, No. 69, Postal Code: 1316943551, Tehran, Iran*

*Email:* [*alimaleki@pasteur.ac.ir*](mailto:alimaleki@pasteur.ac.ir)

Table of Contents

Table S1. Summary of Markov jumps inferred for B.4 dynamics in a discrete phylogeographic analysis to identify introductions through Iran at global level. Posterior mean estimates with 95% HPD intervals. Markov jumps associated with a Bayes factor support higher than 10 corresponding a positive support transition realization and zero values are represented with a hyphen.4

Table S2. Summary of Markov jumps inferred for Alpha dynamics in a discrete phylogeographic analysis to identify introductions through Iran at global level. Posterior mean estimates with 95% HPD intervals. Markov jumps associated with a Bayes factor support higher than 10 corresponding a positive support transition realization and zero values are represented with a hyphen.5

**Table S3.** Summary of Markov jumps inferred for Delta dynamics in a discrete phylogeographic analysis to identify introductions through Iran at global level. Posterior mean estimates with 95% HPD intervals. Markov jumps associated with a Bayes factor support higher than 10 corresponding a positive support transition realization and zero values are represented with a hyphen.8

**Table S4.** Summary of Markov jumps inferred for Omicron dynamics in a discrete phylogeographic analysis to identify introductions through Iran at global level. Posterior mean estimates with 95% HPD intervals. Markov jumps associated with a Bayes factor support higher than 10 corresponding a positive support transition realization and zero values are represented with a hyphen..9

**Table S5.** Summary of Markov jumps inferred for B.4 dynamics in a discrete phylogeographic analysis to characterize the circulation at National level in Iran. Posterior mean estimates with 95% HPD intervals. Markov jumps associated with a Bayes factor support higher than 10 corresponding a positive support transition realization and zero values are represented with a hyphen. 11

**Table S6.** Summary of Markov jumps inferred for Alpha dynamics in a discrete phylogeographic analysis to characterize the circulation at National level in Iran. Posterior mean estimates with 95% HPD intervals. Markov jumps associated with a Bayes factor support higher than 10 corresponding a positive support transition realization and zero values are represented with a hyphen. 13

**Table S7.** Summary of Markov jumps inferred for Delta dynamics in a discrete phylogeographic analysis to characterize the circulation at National level in Iran. Posterior mean estimates with 95% HPD intervals. Markov jumps associated with a Bayes factor support higher than 10 corresponding a positive support transition realization and zero values are represented with a hyphen. 14

**Table S8.** Summary of Markov jumps inferred for Omicron dynamics in a discrete phylogeographic analysis to characterize the circulation at National level in Iran. Posterior mean estimates with 95% HPD intervals. Markov jumps associated with a Bayes factor support higher than 10 corresponding a positive support transition realization and zero values are represented with a hyphen. 15

**Figure S1.** Root-to-tip regressions for all lineages evaluated in this study16

**Figure S2.** The maximum clade credibility (MCC) tree regarding the phylogeographic reconstruction performed for B.4 lineage, with tip colors represented the location of each sequence17

**Figure S3.** The maximum clade credibility (MCC) tree regarding the phylogeographic reconstruction performed for Alpha lineage, with tip colors represented the location of each sequence 18

**Figure S4.** The maximum clade credibility (MCC) tree regarding the phylogeographic reconstruction performed for Delta lineages, with tip colors represented the location of each sequence and shapes are based on sub-lineages presence 19

**Figure S5.** The maximum clade credibility (MCC) tree regarding the phylogeographic reconstruction performed for Omicron lineages, with tip colors represented the location of each sequence and shapes are based on sub-lineages presence 20

| **Table S1.** Summary of Markov jumps inferred for B.4 dynamics in a discrete phylogeographic analysis to identify introductions through Iran at global level. Posterior mean estimates with 95% HPD intervals. Markov jumps associated with a Bayes factor support higher than 10 corresponding a positive support transition realization and zero values are represented with a hyphen. | | | |
| --- | --- | --- | --- |
| Start | Destination | Median | 95% HPD |
| Africa | Africa | - | - |
| Africa | Asia | - | - |
| Africa | Europe | - | - |
| Africa | Iran | - | - |
| Africa | North America | - | - |
| Africa | Oceania | - | - |
| Asia | Africa | 1.88 | 1-2 |
| Asia | Asia | - | - |
| Asia | Europe | 5 | 2-9 |
| Asia | Iran | 8 | 0-16 |
| Asia | North America | 1.42 | 0-4 |
| Asia | Oceania | 5 | 2-9 |
| Europe | Africa | - | - |
| Europe | Asia | - | - |
| Europe | Europe | - | - |
| Europe | Iran | - | 0-3 |
| Europe | North America | - | - |
| Europe | Oceania | - | 0-2 |
| Iran | Africa | - | - |
| Iran | Asia | 59 | 50-69 |
| Iran | Europe | 34 | 29-38 |
| Iran | Iran | - | - |
| Iran | North America | 18 | 14-21 |
| Iran | Oceania | 13 | 10-17 |
| North America | Africa | - | - |
| North America | Asia | - | 0-2 |
| North America | Europe | - | - |
| North America | Iran | - | 0-2 |
| North America | North America | - | - |
| North America | Oceania | - | 0-1 |
| Oceania | Africa | - | 0-6 |
| Oceania | Asia | 1.64 | 0-4 |
| Oceania | Europe | - | 0-1 |
| Oceania | Iran | - | 0-1 |
| Oceania | North America | - | 0-2 |
| Oceania | Oceania | - | - |

| **Table S2.** Summary of Markov jumps inferred for Alpha dynamics in a discrete phylogeographic analysis to identify introductions through Iran at global level. Posterior mean estimates with 95% HPD intervals. Markov jumps associated with a Bayes factor support higher than 10 corresponding a positive support transition realization and zero values are represented with a hyphen. | | | |
| --- | --- | --- | --- |
| Start | Destination | Median | 95% HPD |
| Africa | Africa | - | - |
| Africa | Asia | 4 | 2-6 |
| Africa | Europe | 3 | 0-6 |
| Africa | Iran | - | - |
| Africa | North America | 12 | 8-15 |
| Africa | Oceania | 4 | 2-7 |
| Africa | South America | 4 | 2-7 |
| Asia | Africa | - | - |
| Asia | Asia | - | - |
| Asia | Europe | 1.44 | 0-3 |
| Asia | Iran | 1.23 | 0-4 |
| Asia | North America | 3 | 1-4 |
| Asia | Oceania | 6 | 3-10 |
| Asia | South America | 2 | 0-4 |
| Europe | Africa | 35 | 28-40 |
| Europe | Asia | 39 | 32-45 |
| Europe | Europe | - | - |
| Europe | Iran | 18 | 15-22 |
| Europe | North America | 31 | 26-35 |
| Europe | Oceania | 35 | 27-41 |
| Europe | South America | 37 | 32-43 |
| Iran | Africa | - | - |
| Iran | Asia | - | - |
| Iran | Europe | 1 | 0-2 |
| Iran | Iran | - | - |
| Iran | North America | 4 | 2-5 |
| Iran | Oceania | 4 | 3-6 |
| Iran | South America | - | - |
| North America | Africa | 1 | 0-3 |
| North America | Asia | 1 | 0-2 |
| North America | Europe | 2 | 0-4 |
| North America | Iran | - | - |
| North America | North America | - | - |
| North America | Oceania | 3 | 0-5 |
| North America | South America | 5 | 2-6 |
| Oceania | Africa | - | - |
| Oceania | Asia | 8 | 2-11 |
| Oceania | Europe | 4 | 1-8 |
| Oceania | Iran | 2 | 0-4 |
| Oceania | North America | 1 | 0-3 |
| Oceania | Oceania | - | - |
| Oceania | South America | - | 0-3 |
| South America | Africa | 1 | 0-3 |
| South America | Asia | - | 0-1 |
| South America | Europe | 3 | 0-6 |
| South America | Iran | - | - |
| South America | North America | 2 | 0-4 |
| South America | Oceania | 2 | 0-4 |
| South America | South America | - | - |

| **Table S3.** Summary of Markov jumps inferred for Delta dynamics in a discrete phylogeographic analysis to identify introductions through Iran at global level. Posterior mean estimates with 95% HPD intervals. Markov jumps associated with a Bayes factor support higher than 10 corresponding a positive support transition realization and zero values are represented with a hyphen. | | | |
| --- | --- | --- | --- |
| Start | Destination | Median | 95% HPD |
| Africa | Africa |  |  |
| Africa | Asia | 1 | 0-4 |
| Africa | Europe | 3 | 1-5 |
| Africa | Iran | - | 0-1 |
| Africa | North America | 4 | 2-7 |
| Africa | Oceania | 1 | 0-3 |
| Africa | South America | 4 | 2-7 |
| Asia | Africa | 55 | 46-61 |
| Asia | Asia | - | - |
| Asia | Europe | 57 | 47-65 |
| Asia | Iran | 16 | 12-20 |
| Asia | North America | 65 | 54-72 |
| Asia | Oceania | 51 | 44-56 |
| Asia | South America | 16 | 12-20 |
| Europe | Africa | 3 | 0-7 |
| Europe | Asia | 5 | 0-9 |
| Europe | Europe | - | - |
| Europe | Iran | 7 | 4-10 |
| Europe | North America | 8 | 5-12 |
| Europe | Oceania | 7 | 3-11 |
| Europe | South America | 3 | 0-5 |
| Iran | Africa | 1 | 0-2 |
| Iran | Asia | 4 | 2-6 |
| Iran | Europe | 18 | 15-21 |
| Iran | Iran | - | - |
| Iran | North America | 1 | 0-3 |
| Iran | Oceania | 5 | 2-7 |
| Iran | South America | 6 | 5-7 |
| North America | Africa | 5 | 1-9 |
| North America | Asia | 6 | 2-10 |
| North America | Europe | 5 | 2-8 |
| North America | Iran | 3 | 1-4 |
| North America | North America | - | - |
| North America | Oceania | 6 | 4-9 |
| North America | South America | 8 | 6-10 |
| Oceania | Africa | - | 0-2 |
| Oceania | Asia | - | - |
| Oceania | Europe | - | - |
| Oceania | Iran | 1 | 0-3 |
| Oceania | North America | - | - |
| Oceania | Oceania | - | - |
| Oceania | South America | - | - |
| South America | Africa | - | - |
| South America | Asia | - | - |
| South America | Europe | 3 | 2-5 |
| South America | Iran | - | - |
| South America | North America | 1 | 1-2 |
| South America | Oceania | - | - |
| South America | South America | - | - |

| **Table S4.** Summary of Markov jumps inferred for Omicron dynamics in a discrete phylogeographic analysis to identify introductions through Iran at global level. Posterior mean estimates with 95% HPD intervals. Markov jumps associated with a Bayes factor support higher than 10 corresponding a positive support transition realization and zero values are represented with a hyphen. | | | |
| --- | --- | --- | --- |
| Start | Destination | Median | 95% HPD |
| Africa | Africa | - | - |
| Africa | Asia | 15 | 19-44 |
| Africa | Europe | 87 | 46-126 |
| Africa | Iran | 43 | 12-76 |
| Africa | North America | - | - |
| Africa | Oceania | - | - |
| Africa | South America | - | - |
| Asia | Africa | - | - |
| Asia | Asia | - | - |
| Asia | Europe | 85 | 22-157 |
| Asia | Iran | 81 | 27-150 |
| Asia | North America | - | - |
| Asia | Oceania | 19 | 1-59 |
| Asia | South America | - | - |
| Europe | Africa | 31 | 3-83 |
| Europe | Asia | 117 | 29-213 |
| Europe | Europe | - | - |
| Europe | Iran | 56 | 5-120 |
| Europe | North America | 77 | 25-125 |
| Europe | Oceania | - | - |
| Europe | South America | - | - |
| Iran | Africa | - | - |
| Iran | Asia | 35 | 10-70 |
| Iran | Europe | 132 | 90-176 |
| Iran | Iran | - | - |
| Iran | North America | - | - |
| Iran | Oceania | - | - |
| Iran | South America | - | - |
| North America | Africa | - | - |
| North America | Asia | - | - |
| North America | Europe | 26 | 1-69 |
| North America | Iran | - | - |
| North America | North America | - | - |
| North America | Oceania | - | - |
| North America | South America | 77 | 21-146 |
| Oceania | Africa | - | - |
| Oceania | Asia | 10 | 1-42 |
| Oceania | Europe | - | - |
| Oceania | Iran | 16 | 1-59 |
| Oceania | North America | - | - |
| Oceania | Oceania | - | - |
| Oceania | South America | - | - |
| South America | Africa | - | - |
| South America | Asia | - | - |
| South America | Europe | 19 | 1-56 |
| South America | Iran | 15 | 1-40 |
| South America | North America | 49 | 3-120 |
| South America | Oceania | - | - |
| South America | South America | - | - |

| **Table S5.** Summary of Markov jumps inferred for B.4 dynamics in a discrete phylogeographic analysis to characterize the circulation at National level in Iran. Posterior mean estimates with 95% HPD intervals. Markov jumps associated with a Bayes factor support higher than 10 corresponding a positive support transition realization and zero values are represented with a hyphen. | | | |
| --- | --- | --- | --- |
| Start | Destination | Median | 95% HPD |
| Region 1 | Region 1 | - | - |
| Region 1 | Region 2 | 5 | 4-7 |
| Region 1 | Region 3 | 10 | 8-12 |
| Region 1 | Region 4 | 6 | 5-8 |
| Region 1 | Region 5 | 6 | 5-7 |
| Region 2 | Region 1 |  |  |
| Region 2 | Region 2 | - | - |
| Region 2 | Region 3 | - | - |
| Region 2 | Region 4 | 1 | 0-2 |
| Region 2 | Region 5 | 0 | 0-0 |
| Region 3 | Region 1 | 2 | 1-3 |
| Region 3 | Region 2 | - | - |
| Region 3 | Region 3 | - | - |
| Region 3 | Region 4 | 2 | 1-2 |
| Region 3 | Region 5 | - | - |
| Region 4 | Region 1 | - | - |
| Region 4 | Region 2 | - | - |
| Region 4 | Region 3 | - | - |
| Region 4 | Region 4 | - | - |
| Region 4 | Region 5 | - | - |
| Region 5 | Region 1 | - | - |
| Region 5 | Region 2 | - | - |
| Region 5 | Region 3 | - | - |
| Region 5 | Region 4 | - | - |
| Region 5 | Region 5 | - | - |

| **Table S6.** Summary of Markov jumps inferred for Alpha dynamics in a discrete phylogeographic analysis to characterize the circulation at National level in Iran. Posterior mean estimates with 95% HPD intervals. Markov jumps associated with a Bayes factor support higher than 10 corresponding a positive support transition realization and zero values are represented with a hyphen. | | | |
| --- | --- | --- | --- |
| Start | Destination | Median | 95% HPD |
| Region 1 | Region 1 | - | - |
| Region 1 | Region 2 | 6 | 4-6 |
| Region 1 | Region 3 | 5 | 4-6 |
| Region 1 | Region 4 | 9 | 7-11 |
| Region 1 | Region 5 | 5 | 3-6 |
| Region 2 | Region 1 | - | - |
| Region 2 | Region 2 | - | - |
| Region 2 | Region 3 | - | - |
| Region 2 | Region 4 | - | - |
| Region 2 | Region 5 | - | - |
| Region 3 | Region 1 | - | - |
| Region 3 | Region 2 | - | - |
| Region 3 | Region 3 | - | - |
| Region 3 | Region 4 | - | - |
| Region 3 | Region 5 | - | - |
| Region 4 | Region 1 | - | - |
| Region 4 | Region 2 | - | - |
| Region 4 | Region 3 | - | - |
| Region 4 | Region 4 | - | - |
| Region 4 | Region 5 | 1 | 0-2 |
| Region 5 | Region 1 | - | - |
| Region 5 | Region 2 | - | - |
| Region 5 | Region 3 | - | - |
| Region 5 | Region 4 | - | - |
| Region 5 | Region 5 | - | - |

| **Table S7.** Summary of Markov jumps inferred for Delta dynamics in a discrete phylogeographic analysis to characterize the circulation at National level in Iran. Posterior mean estimates with 95% HPD intervals. Markov jumps associated with a Bayes factor support higher than 10 corresponding a positive support transition realization and zero values are represented with a hyphen. | | | |
| --- | --- | --- | --- |
| Start | Destination | Median | 95% HPD |
| Region 1 | Region 1 | - | - |
| Region 1 | Region 2 | 4 | 3-5 |
| Region 1 | Region 3 | 6 | 5-7 |
| Region 1 | Region 4 | 7 | 4-8 |
| Region 1 | Region 5 | 8 | 5-9 |
| Region 2 | Region 1 | - | - |
| Region 2 | Region 2 | - | - |
| Region 2 | Region 3 | - | - |
| Region 2 | Region 4 | - | - |
| Region 2 | Region 5 | - | - |
| Region 3 | Region 1 | - | - |
| Region 3 | Region 2 | - | - |
| Region 3 | Region 3 | - | - |
| Region 3 | Region 4 | - | - |
| Region 3 | Region 5 | - | - |
| Region 4 | Region 1 | - | - |
| Region 4 | Region 2 | - | - |
| Region 4 | Region 3 | - | - |
| Region 4 | Region 4 | - | - |
| Region 4 | Region 5 | 1 | 1-2 |
| Region 5 | Region 1 | - | - |
| Region 5 | Region 2 | - | - |
| Region 5 | Region 3 | - | - |
| Region 5 | Region 4 | - | - |
| Region 5 | Region 5 | - | - |

| **Table S8.** Summary of Markov jumps inferred for Omicron dynamics in a discrete phylogeographic analysis to characterize the circulation at National level in Iran. Posterior mean estimates with 95% HPD intervals. Markov jumps associated with a Bayes factor support higher than 10 corresponding a positive support transition realization and zero values are represented with a hyphen. | | | |
| --- | --- | --- | --- |
| Start | Destination | Median | 95% HPD |
| Region 1 | Region 1 | 0 | 0-0 |
| Region 1 | Region 2 | 5 | 0-8 |
| Region 1 | Region 3 | - | - |
| Region 1 | Region 4 | 3 | 0-6 |
| Region 1 | Region 5 | 11 | 4-15 |
| Region 2 | Region 1 | - | - |
| Region 2 | Region 2 | - | - |
| Region 2 | Region 3 | - | - |
| Region 2 | Region 4 | - | - |
| Region 2 | Region 5 | 2 | 0-4 |
| Region 3 | Region 1 | - | - |
| Region 3 | Region 2 | - | - |
| Region 3 | Region 3 | - | - |
| Region 3 | Region 4 | - | - |
| Region 3 | Region 5 | - | - |
| Region 4 | Region 1 | - | - |
| Region 4 | Region 2 | - | - |
| Region 4 | Region 3 | - | - |
| Region 4 | Region 4 | - | - |
| Region 4 | Region 5 | 1 | 1-2 |
| Region 5 | Region 1 | - | - |
| Region 5 | Region 2 | - | - |
| Region 5 | Region 3 | - | - |
| Region 5 | Region 4 | - | - |
| Region 5 | Region 5 | - | - |


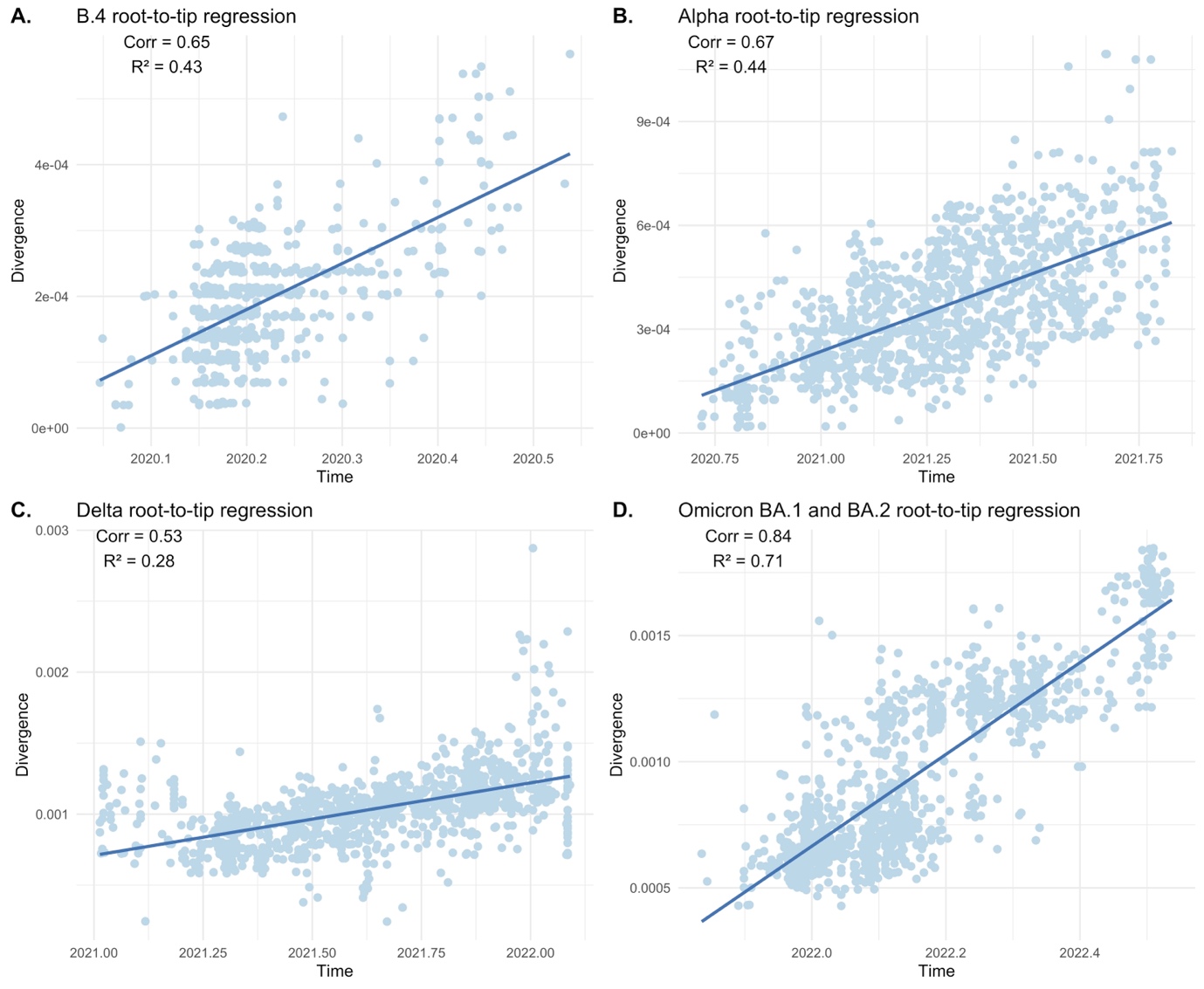


Figure S1. Root-to-tip regressions for all lineages evaluated in this study. The y-axis represents the root-to-tip distances of phylogenetic trees, measured in substitutions per site. In the x-axis shows the time, which each point on the plot represents the tips in the phylogenetic tree. A.B.4. B. Alpha. C. Delta and D. Omicron.


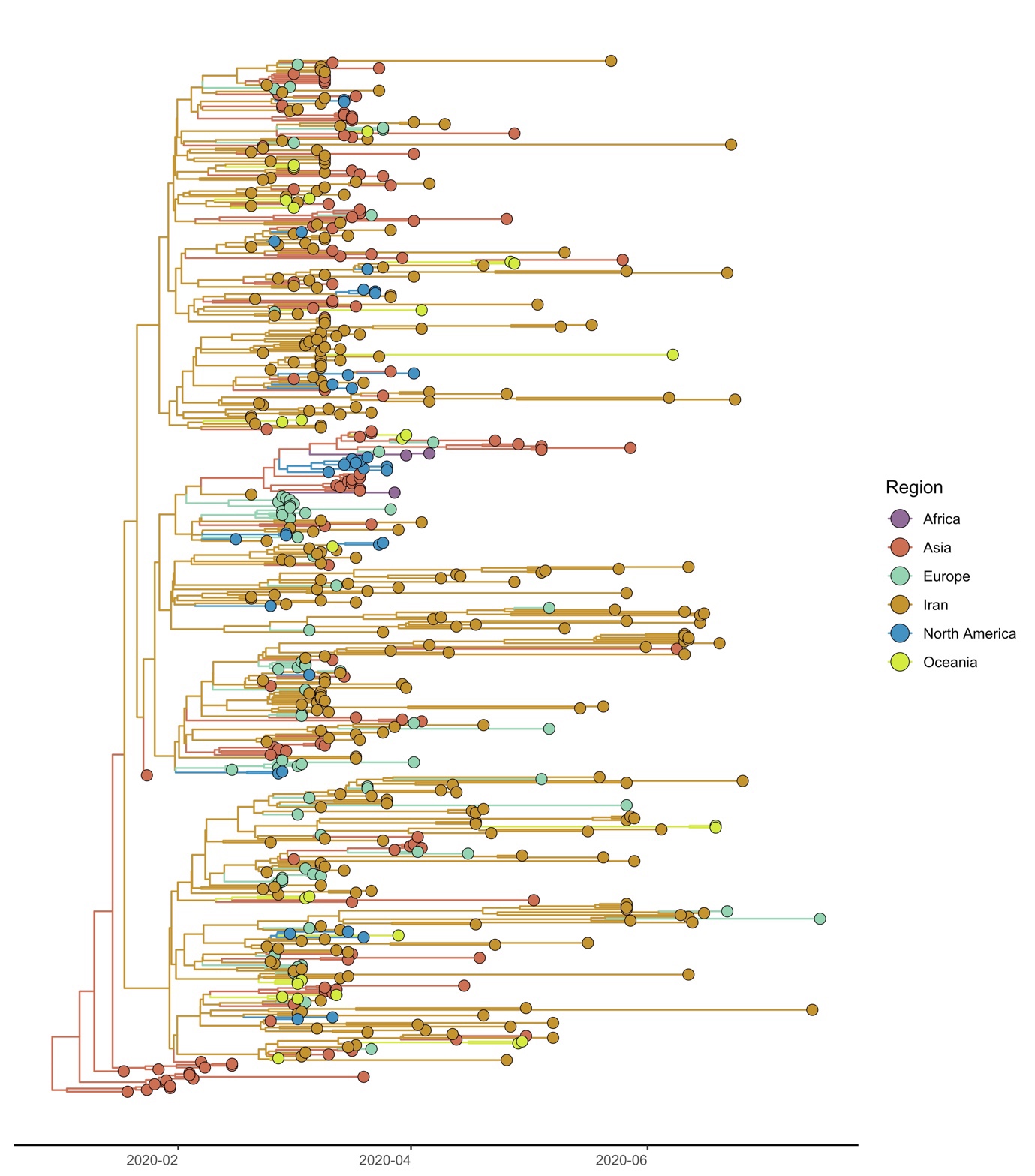


**Figure S2.** The maximum clade credibility (MCC) tree regarding the phylogeographic reconstruction performed for B.4 lineage, with tip colors represented the location of each sequence.


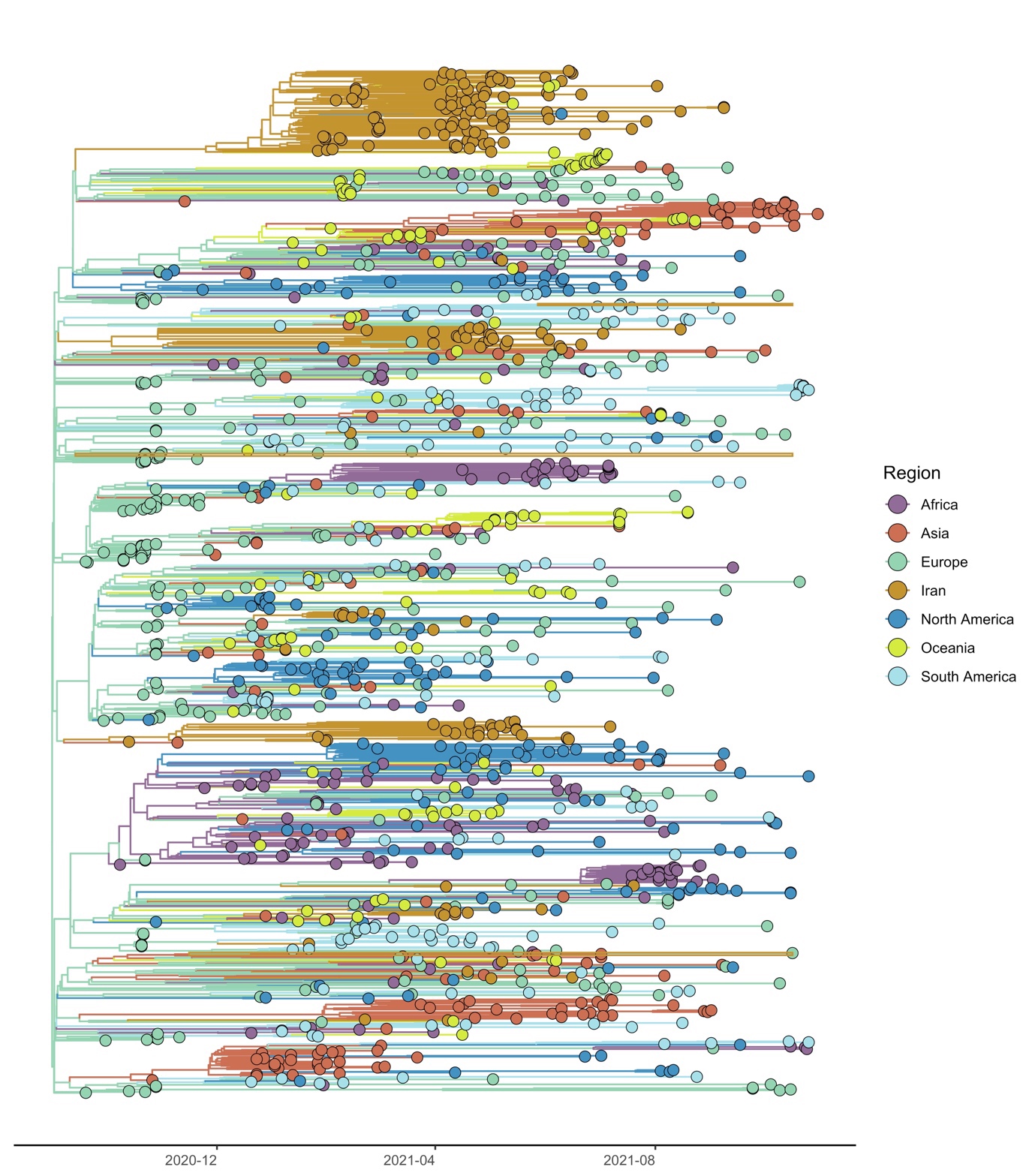


**Figure S3.** The maximum clade credibility (MCC) tree regarding the phylogeographic reconstruction performed for Alpha lineage, with tip colors represented the location of each sequence.


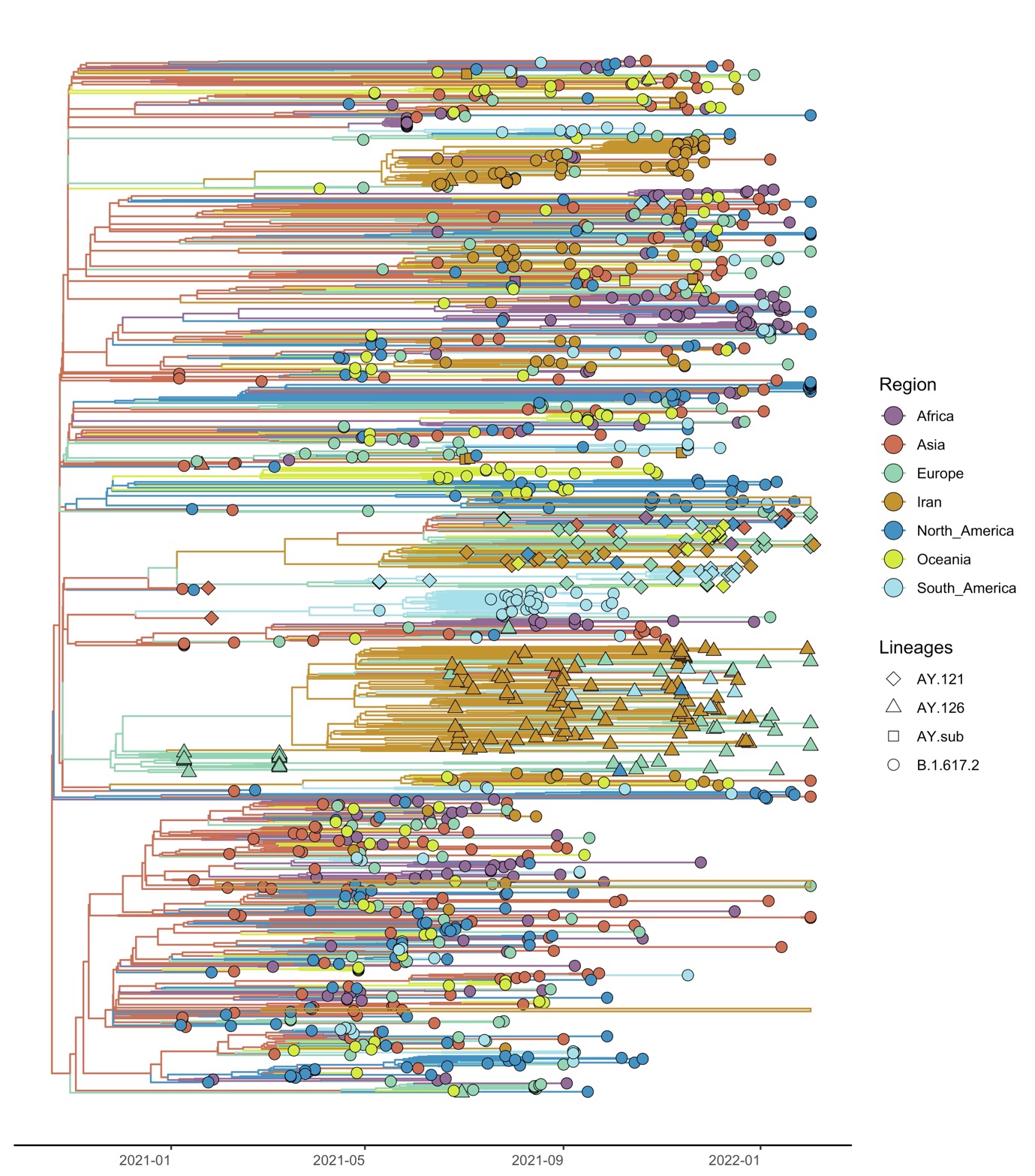


**Figure S4.** The maximum clade credibility (MCC) tree regarding the phylogeographic reconstruction performed for Delta lineages, with tip colors represented the location of each sequence and shapes are based on sub-lineages presence.


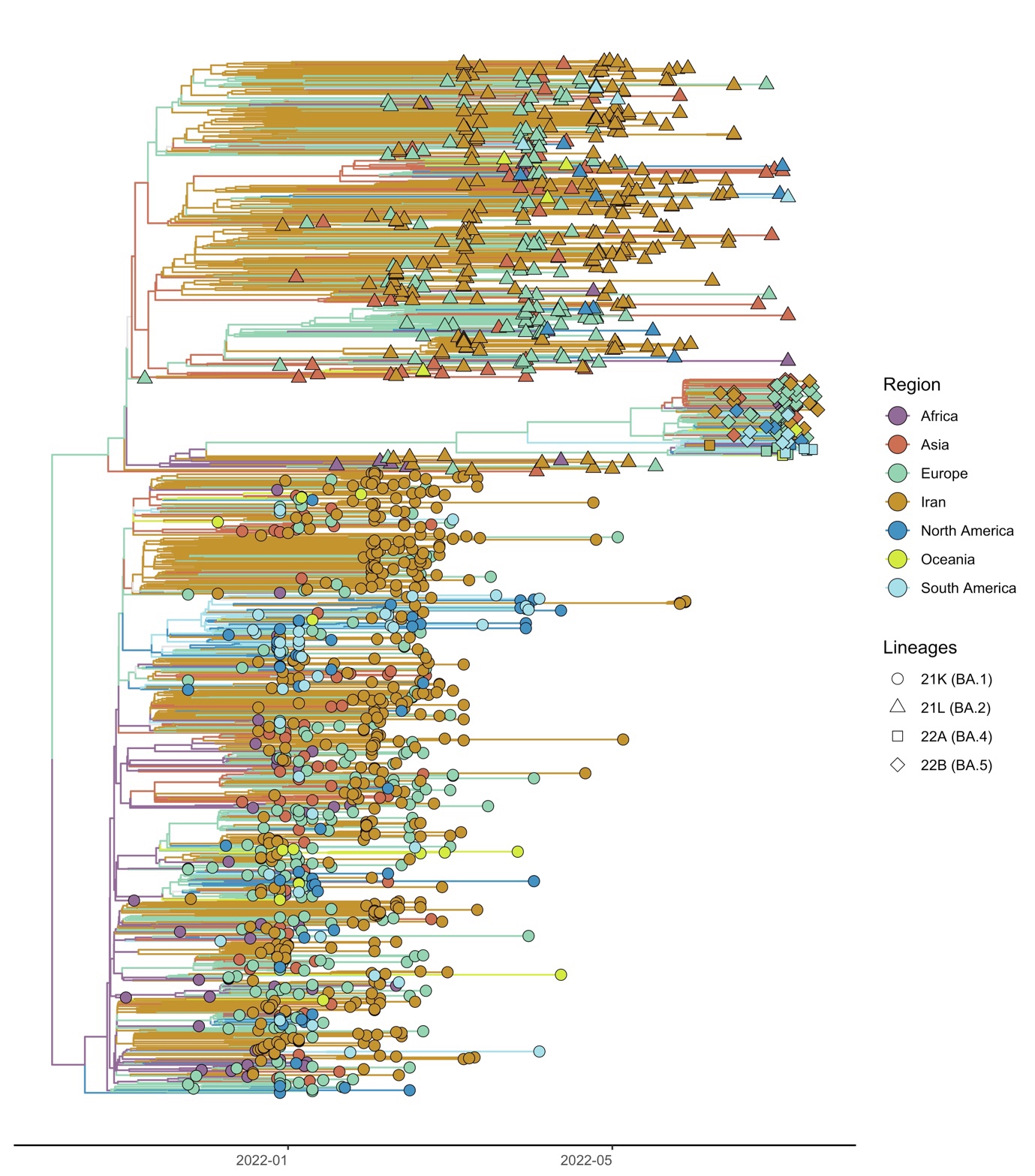


**Figure S5.** The maximum clade credibility (MCC) tree regarding the phylogeographic reconstruction performed for Omicron lineages, with tip colors represented the location of each sequence and shapes are based on sub-lineages presence.
